# Indigestion

**Published:** 1884-06

**Authors:** 


### HALL'S
### JOURNAL OF HEALTH

—AND—

MISCELLANY.

MONTHLY.

E. H. GIBBS, A. M., M. D., EDITOR.

FOUNDED IN 1854.

### INDIGESTION.

INDIGESTION is the source of a multitude of diseases. In fact, disease necessarily follows long continued derangement of the digestive organs. The stomach is the laboratory in which the most wonderful chemical processes are constantly going on. Whatever is taken into the stomach is at once acted upon chemically, and the wholesome and nutritious portions go to build up and strengthen the system, while the effete matters are expelled through the various channels. Now when the food is crude and indigestible, or when good food is not properly masticated and mingled with the saliva before it enters the stomach, it lacks a very important element, and the digesting organs are subjected to great fatigue in efforts to prepare it for its work of sustaining life. When we consider the difficulties with which the stomach has to contend; of improper food forced into the stomach in a crude state and frequently in excess of the requirements of the body; of liquor drinking and tobacco using; the fact that three adult persons out of every four are of unsound health, and that the average term of human life is less than half what it should be under correct methods of living, should surprise no one.

There is not one person in ten who thinks of or seems to care for his health until he loses it.

The most absurd excuse for neglect of duty to oneself is, that he cannot always be looking after his health. In other words, he has not the moral power to abstain from excesses that weaken the body and shorten life, as surely as night follows the day.

What a man eats and drinks habitually has much to do with the condition of mind and body.

The stomach is the fountain-head from whence the body draws its supplies. The old Roman idea of manliness was a "sound mind in a sound body." It is encouraging to know that the more intelligent of the present generation are adopting this view; and it is this class that is now paying the most attention to the laws of health. All colleges encourage to-day what they condemned twenty years ago; as boat racing, ball playing, and regular systems of exercise. If an engineer were to load the safety-valve and build a fire under a steam boiler, and keep the fire replenished for a time without starting the engine, he would produce an explosion. If a man eats heartily, and leads an indolent life, a similar trouble occurs: his body is filled with the products of digestion; fat and fluids accumulate, and the muscles become soft and weak; more blood is made than the system requires, and the excess becomes a dangerous obstruction in the current of life, and the explosion may come in the form of apoplexy. The remedy is, to adapt the quality and the quantity of the food to the daily requirements of the body. Remember that labor, or active and sufficient exercise every day, is imperatively demanded if we expect to keep well. Let the food be plain but nutritious, and avoid rich pastry and other indigestible foods. Adjust the food supply to the bodily activities.

